# The Importance of Stimulus Noise Analysis for Self-Motion Studies

**DOI:** 10.1371/journal.pone.0094570

**Published:** 2014-04-22

**Authors:** Alessandro Nesti, Karl A. Beykirch, Paul R. MacNeilage, Michael Barnett-Cowan, Heinrich H. Bülthoff

**Affiliations:** 1 Department of Human Perception, Cognition and Action, Max Planck Institute for Biological Cybernetics, Tübingen, Germany; 2 German Center for Vertigo and Balance Disorders, Ludwig Maximilians University Hospital of Munich, Munich, Germany; 3 Department of Kinesiology, University of Waterloo, Waterloo, Ontario, Canada; 4 Department of Brain and Cognitive Engineering, Korea University, Seoul, Korea; University of Regensburg, Germany

## Abstract

Motion simulators are widely employed in basic and applied research to study the neural mechanisms of perception and action during inertial stimulation. In these studies, uncontrolled simulator-introduced noise inevitably leads to a disparity between the reproduced motion and the trajectories meticulously designed by the experimenter, possibly resulting in undesired motion cues to the investigated system. Understanding actual simulator responses to different motion commands is therefore a crucial yet often underestimated step towards the interpretation of experimental results. In this work, we developed analysis methods based on signal processing techniques to quantify the noise in the actual motion, and its deterministic and stochastic components. Our methods allow comparisons between commanded and actual motion as well as between different actual motion profiles. A specific practical example from one of our studies is used to illustrate the methodologies and their relevance, but this does not detract from its general applicability. Analyses of the simulator’s inertial recordings show direction-dependent noise and nonlinearity related to the command amplitude. The Signal-to-Noise Ratio is one order of magnitude higher for the larger motion amplitudes we tested, compared to the smaller motion amplitudes. Simulator-introduced noise is found to be primarily of deterministic nature, particularly for the stronger motion intensities. The effect of simulator noise on quantification of animal/human motion sensitivity is discussed. We conclude that accurate recording and characterization of executed simulator motion are a crucial prerequisite for the investigation of uncertainty in self-motion perception.

## Introduction

For more than a century, motion simulators have been employed in neurophysiological, psychophysical and behavioural studies that aim to inform the neural and cognitive processes of self-motion perception [1]–[8], as well as predicting human behaviours such as balance or aircraft control [9]–[11]. In all these studies, motion trajectories executed by the simulator inevitably deviate from the commanded motion. This deviation is due to the mechanics of the device and results in motion distortions that affect amplitudes, frequencies and phases of the commanded trajectories [12]. Throughout this paper we define total noise as the components of the actual motion that are not present in the commanded motion. We further define the total noise as the sum of a deterministic component, reproducible across repetitions of the same trajectory (e.g. mechanical deformations due to the inertia of the simulator), and a stochastic component, representing the random component of the total noise. All sensors, including the human self-motion sensory systems (visual, vestibular, auditory and somatosensory), are frequency dependent (see for example [4], [13]), and signal processing performance can be directly affected by the level of total noise in the system. Moreover, the simulator noise can provide indirect self-motion cues such as velocity dependent vibrations [14]. Response measurements such as neural, perceptual, eye movement or balance recordings should therefore be analysed with a sound understanding of the simulator capabilities and limitations, as well as the impact these limitations have on the results, so as to avoid erroneous interpretations of the data.

While there has been significant prior research on designing, diagnosing and comparing motion systems [12], these methods are not often employed by neuroscientists to dissociate simulator noise from physiological noise in the interpretation of neurophysiological, physiological and behavioural responses. Only a few studies on human self-motion perception address the issue of simulator-introduced noise by recording the actual motion produced. The analyses presented in these perceptual studies can be graphical and/or statistical. In a graphical analysis (see for example [15], [16]), a graphical representation of the simulator’s capability is provided by plotting (in the time and/or frequency domain) the different motion recordings together. A statistical analysis, on the other hand, objectively compares recordings of different motion profiles by applying statistical tests. Note that both statistical and graphical analyses can be used to compare either two different actual motions or commanded versus actual motion. Here we summarize the main statistical approaches used so far to assess the influence of simulator noise on perceptual thresholds for self-motion and we present new methodologies for the noise analysis. These methodologies take inspiration from classic techniques for measuring the dynamic qualities of motion simulators [12] and adapt them where necessary to better dissociate between simulator and physiological noise.

To facilitate the description of the methodologies and their relevance, we will use a psychophysical study conducted by the authors [17]. Briefly, a motion simulator was used to investigate human sensitivity to linear vertical self-motion in a range of 0–2 m/s^2^. Participants were asked to discriminate a reference motion, repeated unchanged for every trial, from a comparison motion, iteratively adjusted in amplitude to measure the participants’ motion discrimination thresholds. Different reference motions were tested in different experimental conditions. When interpreting the experimental results, the undesired noise introduced by the simulator is of concern for two main reasons:

1. The total noise level of reference and comparison motions within each condition, if noticeably different, would provide additional cues to the participants.
2. The increase of the total noise level with motion intensity, if non-linear, would lead to a non-constant Signal-to-Noise Ratio (SNR), resulting in differences in stimulus quality across the tested motion range.

Note that, although different experimental procedures have been proposed and used in the literature to investigate the perception of self-motion, none are immune to these problems.

The first point has been raised already by [18] and by [19]. In each of these studies, motions recorded from an inertial measurement unit (IMU) at different commanded amplitudes were analysed to assess their role in the experiments. In [18] each of their commanded trajectories was recorded multiple times (13 to 19 repetitions) and the averaged signal was subtracted from each trace to isolate the stochastic noise. Note that averaging over many repetitions causes the stochastic noise to decrease with the square root of the number of trials averaged [20], whereas the deterministic component is always present in the average signal no matter how many trials are averaged. The Fourier transform of each trace was then computed to obtain the amplitude-frequency spectrum of the stochastic noise. An ANOVA of the spectra (0.5 to 100 Hz in 0.5 Hz increments) showed no significant differences between profiles. This method provides an objective way to quantify the amount of stochastic noise in each profile by looking at the amplitude spectrum of the frequencies after the average signal is removed. Note that this procedure not only removes the commanded motion signal but also any deterministic component of the total noise. However, if there is reason to believe that the deterministic noise also depends on the motion intensity (e.g. if the amplitude of the deterministic noise increases with the amplitude of the command), the deterministic noise should not be excluded from the motion analysis, as it can provide a noticeable cue.

A different approach, employed by [19], suggests comparing two different stimuli by treating the two digital IMU measures as two different distributions after the commanded signal is filtered out in the frequency domain. A t-test between these two distributions is used to show that the amount of total noise is not significantly different. Because the t-test is specifically designed to compare the means of two populations, this method is able to detect changes in the total noise mean but remains insensitive to changes in the total noise amplitude (the distribution extremes) as long as the two signals have similar means. It is however reasonable to expect that the end-effector of the simulator oscillates around the desired trajectory yielding mean simulator noise close to zero for every trajectory. On the other hand, any effect of motion intensity on the amplitude of the noise will not be detected. For this reason, we did not apply this methodology in the present work.

To the best of our knowledge, the second point, concerning changes in the signal quality across the tested motion intensity range, has never been addressed in any psychophysical study on self-motion perception. Substantial evidence indicates that the SNR of motion simulators depends on the commanded motion intensity and frequency (cf. [12], [21]) and that human self-motion sensitivity is affected by stimulus SNR [22]. Therefore, we believe that signal quality is a potential confound in the analysis of self-motion responses and should always be given careful consideration. It is not our goal here to investigate the effect of the motion SNR on human self-motion sensitivity. Instead we present an SNR analysis of the motion profiles, which constitutes an essential step for a correct interpretation of experimental results.

For our chosen example study [17], it is most appropriate to analyse the total noise because each trial consisted of both a reference and comparison motion, of unequal amplitude, leading to potential differences in both deterministic and stochastic noise. Using total noise is best when the deterministic component may alter the results. However, for comparison, here we also present methodologies to quantify the relative contribution of stochastic and deterministic components and their dependencies on the commanded motion. Separate analysis of deterministic and stochastic components is relevant, for example, in studies where many repetitions of the same command are employed (e.g. for measuring gains of the vestibular ocular reflex). In these cases the actual motion stimulus is the motion command combined with the deterministic noise, and deviations of eye traces from the actual motion command are caused by physiological noise (focus of interest) and stochastic noise (undesired simulator-introduced variability).

## Methods

**Figure 1:**
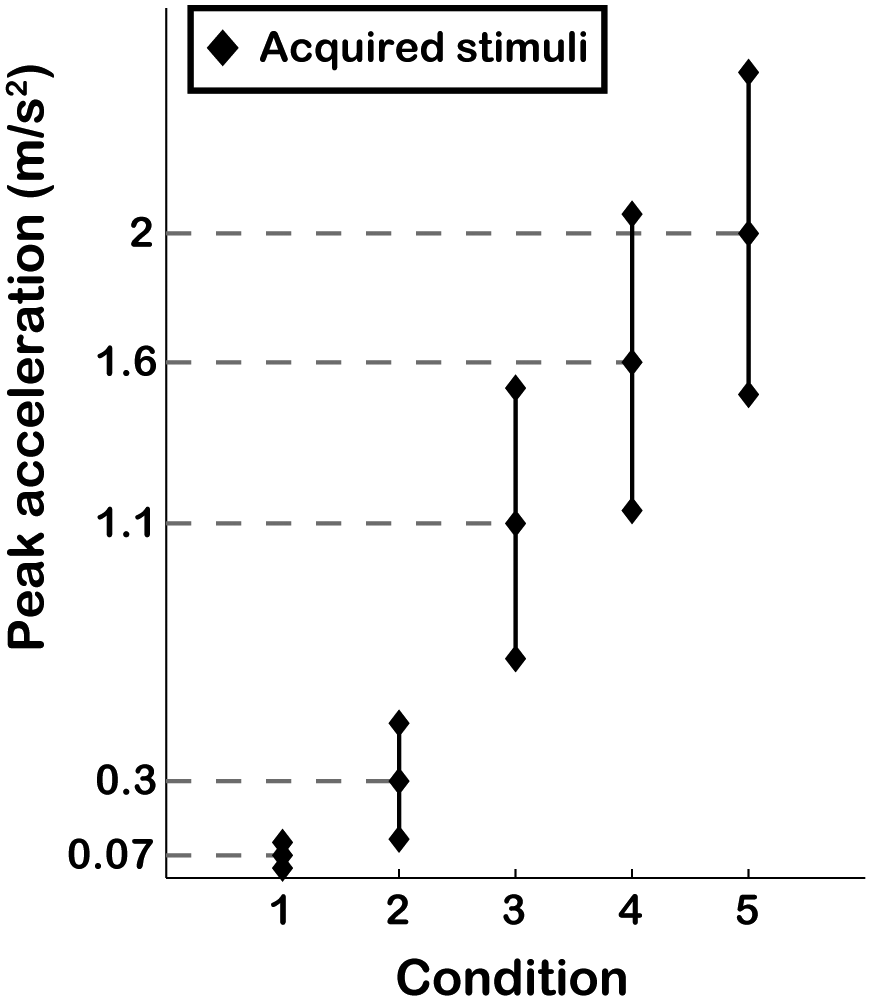
Acquired stimuli. Graphical representation of peak amplitude for the acquired stimuli for both upward and downward motion. The dashed lines indicate the reference intensities, around which the higher and lower comparison were set.

**Figure 2:**
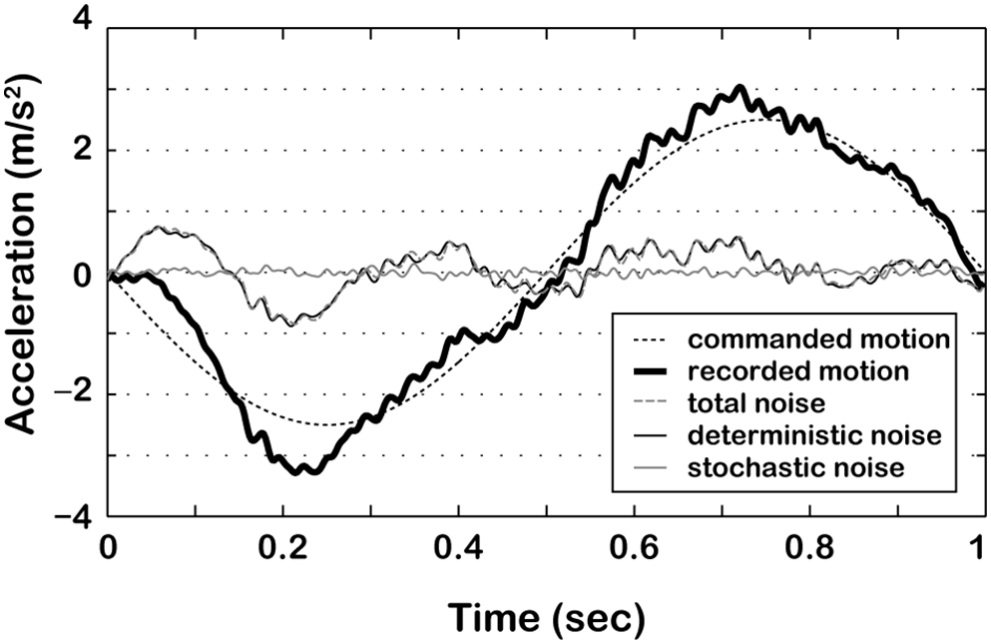
Example of an acquired motion profile and its component. The total noise (grey dashed line) was obtained by subtracting the input command (black dashed line) from the acquired acceleration profile after low-pass filtering (black thick line). The figure also illustrates the deterministic (black thin line) and stochastic (grey thin line) components of the total noise of the recorded profile.

The study was conducted using the Max Planck Institute CyberMotion Simulator, a 6-degrees-of-freedom anthropomorphic robot-arm, able to provide a large variety of motion stimuli, with a maximal vertical displacement of about 1.4 m and a maximal vertical linear acceleration of about 5 m/s^2^ (for technical details refer to Robocoaster, KUKA Roboter GmbH, Germany; [23], [24]). IMU traces were acquired for 10 reference stimuli (1 Hz sinusoidal acceleration profiles with peak amplitudes of 0.07, 0.3, 1.1, 1.6 and 2 m/s^2^, both upward and downward) with a 3D accelerometer (YEI 3-Space Sensor, 500 Hz) attached rigidly on the back of the simulator seat. While our trajectories did not involve rotations, it is important to note that for rotational trajectories, seat and head motions differ and placing the IMU on the participant’s head is a more sensible choice. For each reference stimulus, we additionally recorded two comparison stimuli whose peak intensity was raised (higher comparison) and lowered (lower comparison) by two corresponding discrimination thresholds, so as to quantify the noise level changes within stimuli of the same condition. The discrimination thresholds associated with the reference stimuli are 0.02 (unpublished observation), 0.09, 0.21, 0.23 and 0.25 m/s^2^, respectively [17]. Each profile (Fig. 1) was recorded 20 times. Of the recorded profiles, only the frequency components below 80 Hz were considered for further analyses, under the assumption that for these profiles frequencies higher than 80 Hz do not affect psychophysical performance (in agreement with [19]). From each signal the 1 Hz input was subtracted to obtain the total noise signal. Fig. 2 illustrates the procedure for a downward acceleration with peak amplitude of 2.5 m/s^2^. The deterministic component was obtained by averaging the total noise across repetitions of each profile, and the stochastic component was obtained by subtracting the deterministic component from the total noise.

Two different methods were employed to analyse the total noise level of the profiles: the amplitude-frequency spectrum and the root mean square (rms). These methods are explained in more details in the following sections. Additionally, for the 10 reference stimuli, the SNR was computed to characterize the relationship between the quality of the reproduced motion and the intensity of the commanded motion (section Signal-to-noise ratio analysis). We further analyse these stimuli in terms of the deterministic and stochastic components of their total noise (section Deterministic and stochastic noise analysis). Signal processing and statistical analysis were performed in MATLAB (2012a) using custom-written code and the Statistics toolbox.

### Amplitude-frequency Spectrum Analysis

The total noise affecting the motion profiles can be objectively quantified by its amplitude-frequency spectrum. Such an indicator has the advantage of providing details about which frequencies are more affected by the noise. This approach, based on [18], differs from the original work described previously since from each acquired trace only the input command is removed, rather than the average over several repetitions (which contains both input and deterministic noise). This allows for an analysis of the total rather than the stochastic noise. Force/exponential windows of one and two seconds, respectively, were applied to the original signals according to equation 1. This allows for reduction of frequency leakage [25], [26] without altering the amplitude of the total noise signal contained in the first second of the window.
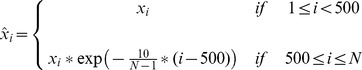
(1)where x_i_ hat is the i-th sample of the windowed signal, x_i_ is the i-th sampled measure of noise and N is the number of samples (in this case 1500 samples).

**Figure 3:**
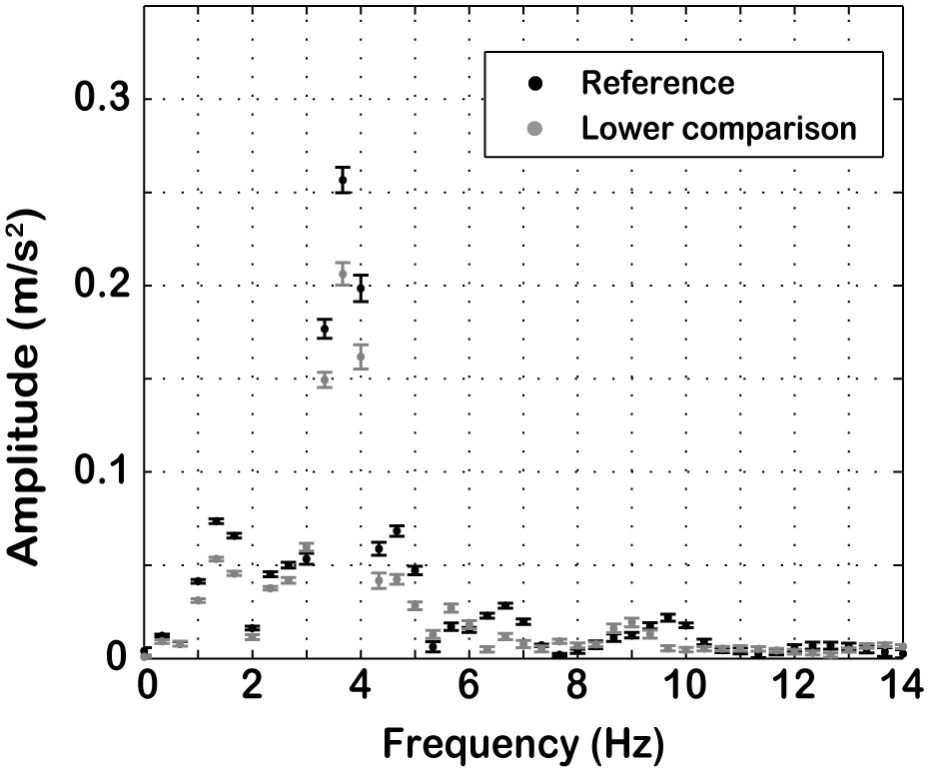
Amplitude-frequency spectrum. Example of an amplitude-frequency spectrum for a reference of 1.6 m/s^2^ and corresponding lower comparison. In this example the simulator noise mainly affects frequencies around 4 Hz. Error bars represent standard deviations of the 20 repetitions of each profile. The abscissa is limited to only the first 61 frequency components (0–20 Hz) out of the 241 (0–80 Hz) used for the analysis for better graphical clarity.

After Fourier transforming the windowed signals we obtained 3 groups of 20 amplitude-frequency spectra for each condition (Fig. 1): one group for the reference motion, one for the higher comparison and one for the lower comparison. A typical amplitude-frequency spectrum is presented in Fig. 3. Note that it is possible to infer the main frequencies that compose the total noise from spectral analysis. The 20 amplitude spectra of each reference motion were tested against the 20 amplitude spectra of their corresponding higher and lower comparisons independently by using an ANOVA with 2 factors: frequency (0 to 80 Hz in 0.33-Hz increments, yielding 241 values) and motion profile (reference or comparison).

The same analysis was also performed on the stochastic noise so as to allow for comparison with the *analysis of vibration* reported in [18] (note the change in terminology from “vibration” to “stochastic noise”). Results of these analyses are shown in Table 1.

**Table 1:** Statistical differences in compared profiles.

Compared profiles (referencevs. comparison)	rms analysis				Amplitude–frequency spectrum analyses			
[m/s^2^]	[t(38), p values]				[df = 1, p values]			
	Total Noise		Stochastic Noise		Total Noise		Stochastic Noise	
	up	down	up	down	up	down	up	down
0.07 vs. 0.03	**<0.001**	**<0.001**	0.47	0.68	**<0.001**	**<0.001**	**<0.001**	0.06
0.07 vs. 0.11	**<0.001**	**<0.001**	0.66	0.11	**<0.001**	**<0.001**	**0.03**	**<0.001**
0.3 vs. 0.12	**<0.001**	**<0.001**	0.05	**0.02**	**<0.001**	**<0.001**	**<0.001**	**<0.001**
0.3 vs. 0.48	**<0.001**	**<0.001**	0.18	**0.002**	**<0.001**	**<0.001**	**0.02**	**<0.001**
1.1 vs. 0.68	**<0.001**	**<0.001**	0.39	0.57	**<0.001**	**<0.001**	**<0.001**	**<0.001**
1.1 vs. 1.52	**<0.001**	**<0.001**	0.98	0.38	**<0.001**	**<0.001**	0.11	**0.002**
1.6 vs. 1.14	**<0.001**	**0.005**	0.36	0.06	**<0.001**	**<0.001**	**<0.001**	**<0.001**
1.6 vs. 2.06	**<0.001**	**<0.001**	0.38	0.19	**<0.001**	**<0.001**	**<0.001**	**<0.001**
2 vs. 1.5	**<0.001**	**<0.001**	0.08	**<0.001**	**<0.001**	**<0.001**	**<0.001**	**<0.001**
2 vs. 2.5	0.12	**<0.001**	0.21	**0.01**	**<0.001**	**<0.001**	**<0.001**	**<0.001**

P-values resulting from the two analyses comparing the levels of total and stochastic noise around each reference for both upward (up) and downward (down) movements. Effects with a p-value <0.05 are considered as significant and appear in bold.

### Root Mean Square Analysis

The rms, or quadratic mean, is a measure of the magnitude of a varying quantity. Here, its discrete formula is used to objectively quantify the noise level of each 1 sec signal:
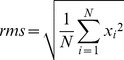
(2)where *x_i_* is the i-th sampled measure of noise and *N* is the number of samples (in this case 500 samples). For each condition we obtained 3 groups of 20 rms values each: one group for the reference motion, one for the higher comparison and one for the lower comparison, which were all repeated 20 times. To determine whether noise level changes within conditions are reliable cues for motion amplitude discrimination, every reference rms group was tested for statistically significant differences (unpaired 2-sample t-test) against its corresponding higher and lower comparison independently. This rms analysis was conducted on the total noise as well as the stochastic component of the noise. Results of these analyses are shown in Table 1.

### Signal-to-Noise Ratio Analysis

The SNR is used to express the relative amount of commanded signal and background noise present in each trajectory. A SNR close to 1 indicates that the level of noise in the reproduced motion is comparable to the level of commanded signal. This is often the case for motion simulators when reproducing small accelerations (e.g. <10 cm/s^2^ for the simulator tested here) [21], [27]. Higher SNRs indicate a reproduced motion of higher quality, where the signal level overcomes the noise. We computed the SNR for every repetition of the 10 reference motions according to the following formula:
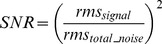
(3)


**Figure 4:**
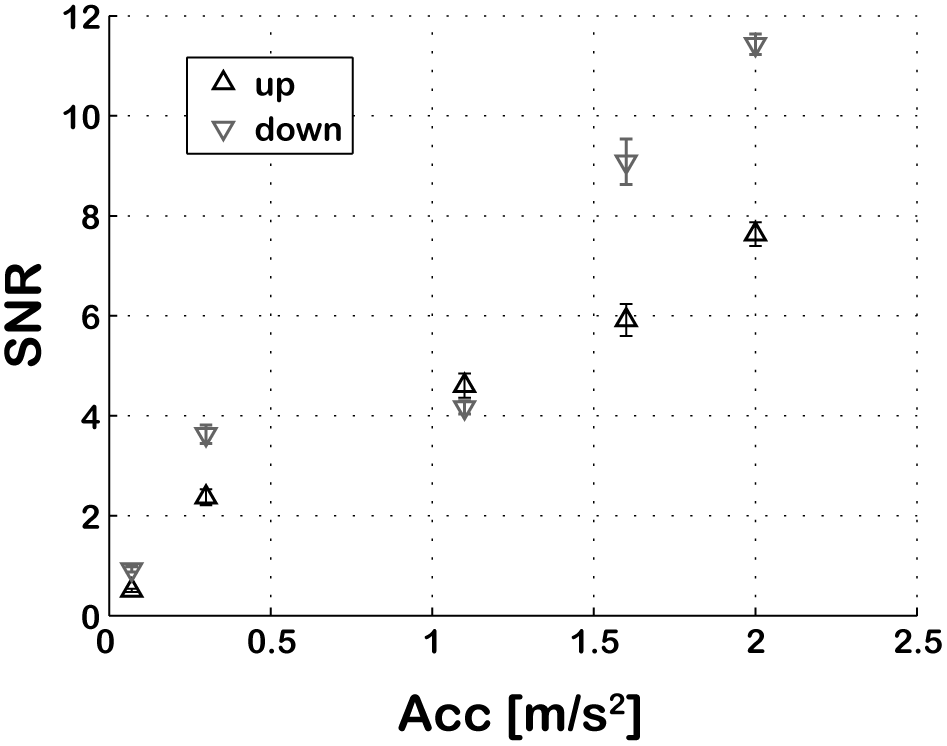
SNRs for motion profiles. The SNR for upward and downward motion profiles increases as a function of motion intensity. Error bars represent standard deviations of the 20 repetitions of each profile.

Differences in the SNRs were tested with an ANOVA with 2 factors: direction (upward or downward) and motion intensity (0.07, 0.3, 1.1, 1.6 and 2 m/s^2^). See Fig. 4 for results.

### Deterministic and Stochastic Noise Analysis

**Figure 5:**
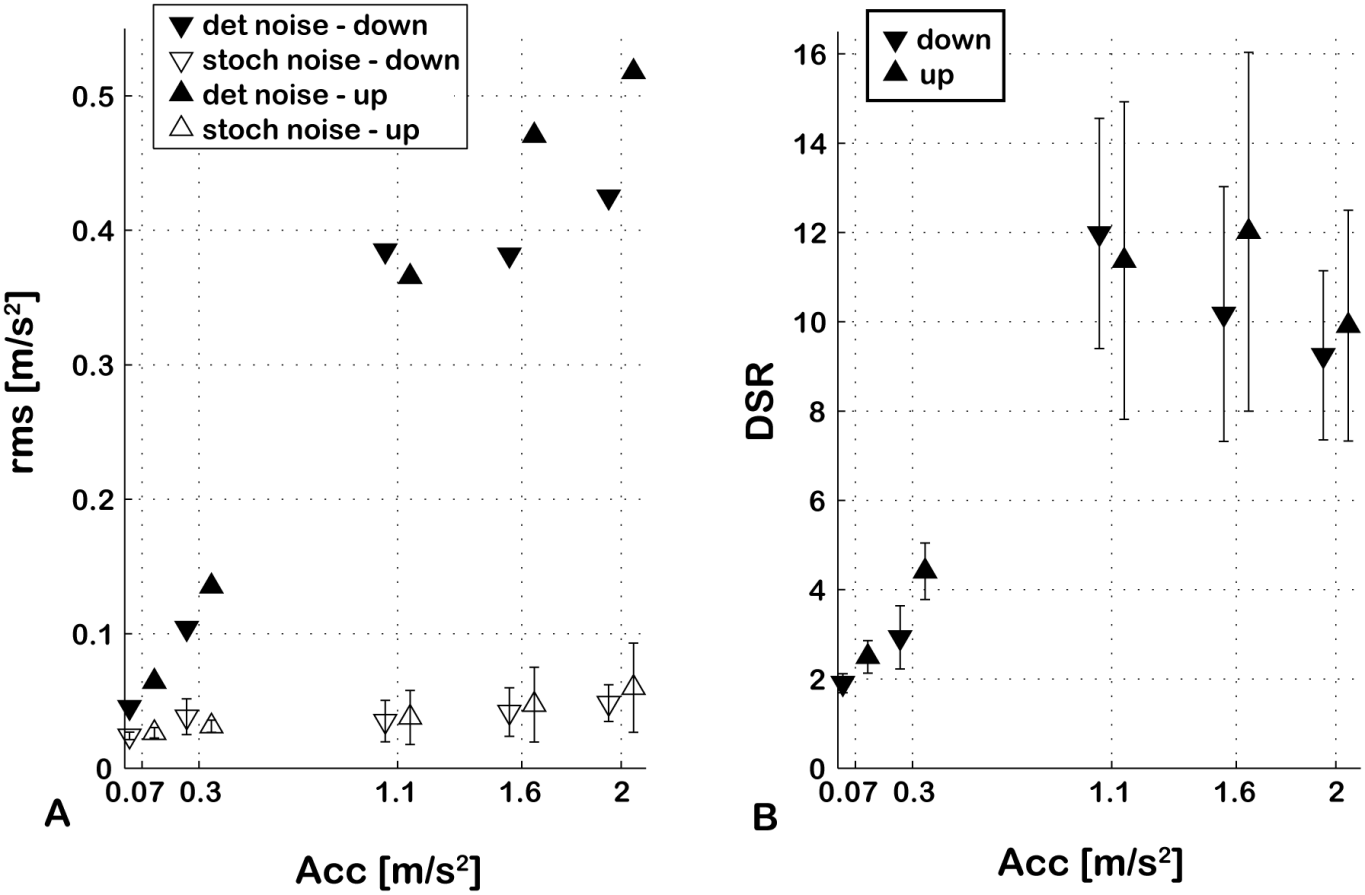
Relative contributions of deterministic and stochastic components to the total noise. **Panel A:** Each reference stimulus recording is associated with the rms of its deterministic (filled triangles) and stochastic component (empty triangles) for both upward (upward pointing triangles) and downward (downward pointing triangles) motions. **Panel B:** The DSR for upward and downward profiles. Both panels indicate a predominance of deterministic over stochastic noise in the recorded profiles. Error bars represent standard deviations of the 20 repetitions of each profile.

Quantitative measures of the stochastic and deterministic noise components in a reproduced motion allow for characterization of the nature of the noise, perhaps providing important information for deciding how to deal with the noise (see discussion). We calculated the rms of the stochastic and deterministic noise for the 10 reference motions. The Deterministic-to-Stochastic Ratio (DSR) introduced in equation 4 indicates which component is dominant in an analysed profile and the way that the total noise composition changes over different stimulus intensities. The results are presented in Fig. 5.
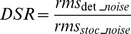
(4)


### Instrumentation and Environmental Noise

Even when recording no motion, a certain level of background activity is to be expected in any IMU recording. This is due to electrical interferences as well as specific traits of the IMU, which obviously do not reflect real motion. New software and hardware improvements are continuously being developed to reduce this sensor noise (see e.g. [20], [28]), however it can never be completely eliminated. The analyses proposed in this manuscript assume that sensor noise, in comparison to motion noise, is negligible. This is often the case for high quality sensors, where sensor shielding strongly reduces electrical interferences. However, in this work the demonstration of the proposed techniques was done using commercial hardware, potentially sensitive to environmental noise such as electrical interferences from the simulator motors. To quantify the level of sensor noise affecting the recordings, IMU data were acquired for approximately 4 minutes while the motion platform was powered but not moving and the rms of the acceleration signal was calculated according to equation 2.

## Results

The tested reference/comparison pairs show significantly different total noise levels both in terms of amplitude-frequency spectrum and rms of the total noise signals in almost every tested pair (table 1). This indicates that the total noise introduced by the simulator depends on the commanded motion intensity.

As expected, the stochastic noise (quantified by its rms value) correlates with the inverse of the square root of the number of trials averaged (in all groups 0.77≤r≤0.99, average r = 0.89). However, sensor noise analysis revealed comparable noise levels between the no-motion profile (rms mean +/− std:  = 0.05+/−0.008 m/s^2^) and the motion profiles (see Fig. 5A). This suggests that stochastic noise is likely to reflect predominantly sensor noise rather than real motion noise, such that the level of stochastic noise in the recorded trajectories is impossible to resolve with the current equipment. Results from the amplitude-frequency spectrum analysis and the rms analysis performed on the stochastic noise alone (see table 1) should therefore be interpreted with caution, as they likely reflect features of the sensor noise rather than physical motion. The overall level of deterministic noise in the motion trajectories (see Fig. 5A) is instead often higher than the sensor noise rms, making it unlikely for the sensor noise to significantly influence the analyses of the deterministic and total noise components.

The total noise rms was found to increase non-linearly with the amplitude of the commanded reference signal (F(4,199) = 25037, p<0.001) over the tested range, which leads to SNRs that depend on the motion intensity (Fig. 4). The results show SNRs one order of magnitude higher for the stronger than for the weaker measured profiles. Moreover, SNRs were overall better for downward compared to upward motion (F(1,199) = 1560, p<0.001). These results suggest that for perceptual systems whose sensitivity increases with SNRs, regardless of motion intensity, motion discrimination using this particular simulator should be proportionally better for downward as compared to upward motions and for higher as compared to lower motion intensities. Experimental data on human motion sensitivity over wide motion ranges [15], [17], [19], [27], however, do not show such behaviour, suggesting an additional noise source inherent to the perceptual system which is proportional to stimulus intensity. Asymmetries in vertical motion sensitivity [17], instead, might be entirely explained by the results of the SNR analysis.

The rms of the stochastic and deterministic noise of each reference profile for upward and downward movements is presented in Fig. 5A. The level of the deterministic noise increases notably with the stimulus intensity and overall is higher than the level of the stochastic noise, which on the other hand remains rather constant over the tested motion range. Consequently, DSRs increase for stronger motion intensities. Note that, as stated above, the stochastic components of the motion noise are likely lower than the noise of the employed sensor. Therefore, the rms of the stochastic noise represents an “upper bound” for the true rms value of the stochastic noise in the motion profiles. Nevertheless, from these results emerges a dominance of the deterministic component of the total noise over the stochastic component, particularly at the higher motion intensities. This suggests that deterministic noise is more likely than stochastic noise to impact self-motion perception in this experimental paradigm.

## Discussion

The analyses presented here allow characterization of the noise introduced by the simulator when reproducing commanded trajectories. They provide sensitive methods to compare the noise level of different commanded stimuli and to graphically and statistically describe the reproduced motion. Results show that the total noise of the simulator increases with the amplitude of the command in a nonlinear way, leading to an SNR that increases with the motion intensity. Even for relatively small changes in the amplitude of the commanded motion, changes in the measured noise are statistically significant. This raises the question of whether a human, when asked to report changes in the motion intensity, could use changes in the simulator noise as a cue rather than changes in the signal itself.

It is reasonable to assume that, if our analyses of the IMU signals do not demonstrate these differences, the human will also not detect them. It is however erroneous to conclude the opposite. The simulator motion is available to the CNS only after being processed by the sensory systems (vestibular, somatosensory and proprioceptive), whose dynamics are imperfect due to frequency dependences and noise [29], [30]. Furthermore, the way that the CNS deals with these signals is likely different from the statistical analysis employed here. Consider as an example the mp3 and AAC encoding techniques in music: even though the frequency spectra of the original and compressed signals look dramatically different, they are virtually indistinguishable to a human observer due to the inability of the auditory system to perceive the differences [31]. Although the frequency response range of the otolith organs of the vestibular system is estimated to be between 0 Hz and 1.6 Hz [32], the contribution of the other sensory systems should not be neglected. For this reason we did not filter the data with a model of the vestibular system.

Given these results and the previous considerations, speculations can be made as to how simulator noise affects human perception of motion intensity. To distinguish between motions at different intensities, differences in the neuronal signals that reach the CNS need to overcome the internal noise level [33]–[35]. If the internal noise is small relative to the total noise of the motion, motion stimuli with high SNR are likely to generate neuronal signals that also have a high SNR. This would facilitate the process of detecting changes in the motion intensity. Additionally, human self-motion sensitivity could be enhanced by changes in the motion noise level if those changes are captured by the human sensors and successfully processed by the CNS. An accurate analysis of the noise of the experimental setup is therefore of great importance for the active research field investigating the noise in the nervous system and its effect on information processing [36].

By including a demonstration of the proposed methodologies in this paper, we raise the practical concern of sensor noise, which affects any measuring setup regardless of the sensor nature (e.g. IMUs, optical trackers, etc.). Sensor measures during no motion allowed us to estimate the level of sensor noise and to conclude that stochastic components of the noise motion are likely smaller than the level of sensor noise, indicating that simulator-introduced noise is primarily of deterministic nature. A more precise quantification of the stochastic motion noise was precluded by the stochastic sensor noise. Overall, caution is advised in the interpretation of sensor measurements as much as in the interpretation of responses to noisy self-motion stimuli.

**Figure 6:**
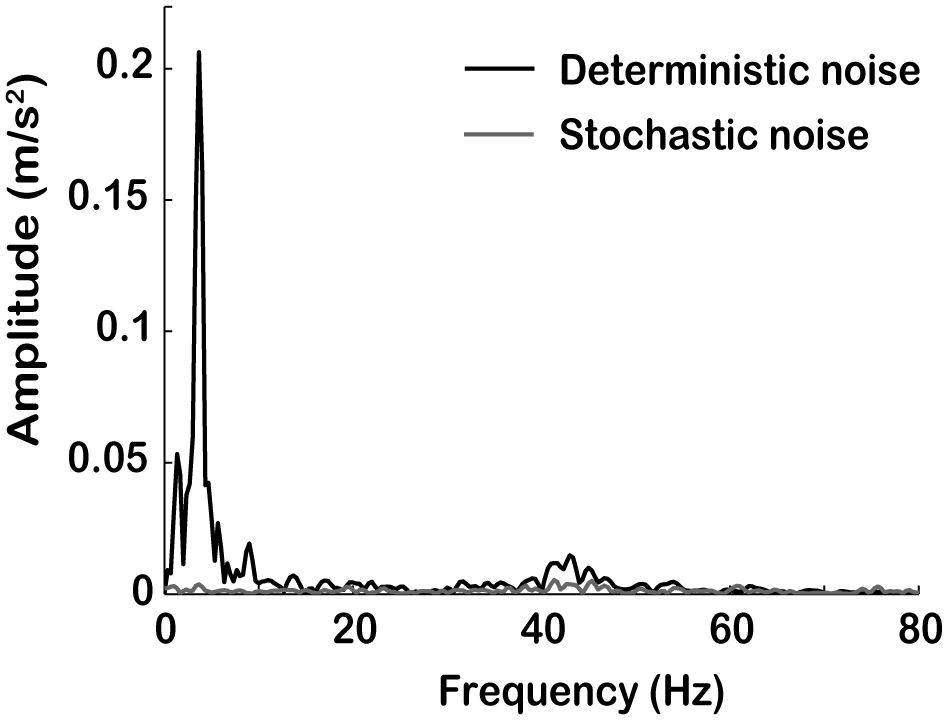
Amplitude-frequency spectrum of the deterministic and stochastic noise components. Amplitude-frequency spectrum of the deterministic and stochastic noise components of the acceleration profile whose total noise is illustrated in Fig. 3. The DSR of this profile is 13.48.

To address the stochastic and deterministic composition of the total noise, we have provided their formal definitions and a methodology for extracting them from IMU recordings. Other than using the DSR introduced here, deterministic and stochastic noise can also be compared using a frequency analysis, which is particularly useful for highlighting the frequency ranges affected by the two types of noise (see the examples in Fig. 6).

Whenever possible effort should be spent in minimizing the deterministic noise, so that its impact during experiments is also minimized. This is particularly beneficial in cases where a DSR analysis indicates a predominantly deterministic nature of the noise. Deterministic noise can be reduced by using iterative learning control algorithms [37], [38]: given a desired trajectory these algorithms iteratively process IMU recordings of the simulator motion and modify the simulator commands so as to track the desired trajectory as closely as possible.

## Conclusion

Simulator-introduced noise is a recurrent concern for neuroscientists who use motion simulators to investigate the neural and cognitive mechanisms of self-motion perception. In this work we developed straightforward graphical and statistical techniques for the analysis of motion stimuli commonly employed in self-motion studies. Rather than measuring the dynamic qualities of a motion system, these analyses allow for dissociation between simulator and physiological noise and therefore constitute a valuable set of tools for the interpretation of neurophysiological and behavioural responses, as well as for meaningful comparisons across the existing literature. We further illustrated these analyses and their relevance using a prior study on human self-motion perception. Results clearly demonstrate the importance of noise, including both stochastic and deterministic components. It should be noted that, although the methods are of general application, the presented results hold for the employed simulator only and other simulators are expected to show substantial differences in their dynamic responses.
